# Use of bladder antimuscarinics is associated with an increased risk of dementia: a retrospective population-based case–control study

**DOI:** 10.1038/s41598-021-84229-2

**Published:** 2021-03-01

**Authors:** Tomor Harnod, Yu-Cih Yang, Lu-Ting Chiu, Jen-Hung Wang, Shinn-Zong Lin, Dah-Ching Ding

**Affiliations:** 1 Department of Neurosurgery, Hualien Tzu Chi Hospital, Buddhist Tzu Chi Medical Foundation, Tzu Chi University, Hualien, Taiwan, ROC; 2 Management Office for Health Data, China Medical University Hospital, Taichung, Taiwan, ROC; 3 College of Medicine, China Medical University, Taichung, Taiwan, ROC; 4 Department of Research, Hualien Tzu Chi Hospital, Buddhist Tzu Chi Medical Foundation, Tzu Chi University, Hualien, Taiwan, ROC; 5 Department of Obstetrics and Gynecology, Hualien Tzu Chi Hospital, Buddhist Tzu Chi Medical Foundation, Tzu Chi University, No. 707, Chung-Yang Rd., Sec. 3, Hualien, Taiwan, ROC; 6 Institute of Medical Sciences, Tzu Chi University, Hualien, Taiwan, ROC

**Keywords:** Bladder disease, Dementia

## Abstract

The association between bladder antimuscarinic use and dementia development is unclear. We used data from the Taiwan National Health Insurance Research Database to determine the association between the exposure dose and duration of bladder antimuscarinics and the subsequent dementia risk. We enrolled participants aged 55 years or more and defined a dementia cohort (International Classification of Diseases, Ninth Revision, Clinical Modification codes 290, 294.1, and 331.0). We used a propensity score matching method, and randomly enrolled two controls without dementia. We evaluated dementia risk with respect to the exposure dose and duration of treatment with seven bladder antimuscarinics (oxybutynin, propiverine, tolterodine, solifenacin, trospium, darifenacin, and fesoterodine) used for at least 1 year before the index date, after adjusting for age, sex, comorbidities, and medications. The dementia risk was 2.46-fold (95% confidence interval: 2.22–2.73) higher in Taiwanese patients who used bladder antimuscarinics for ≥ 1 year than in those who were not exposed to this treatment. The risk proportionally increased with increasing doses of antimuscarinics for less than 4 years. Taiwanese patients aged 55 years or more on bladder antimuscarinics exhibited a higher risk of dementia. Additional studies in other countries are required to determine whether this result is valid worldwide.

## Introduction

Dementia is a common neurological degenerative disorder, the prevalence of which increases with age. Dementia is currently one of the leading causes of disability and death in older adults, and there are no reliable treatments to reverse the development and progression of dementia. However, some evidence suggests that changing the lifestyle and environment of patients may help reduce dementia development^1,2^. Consequently, dementia is usually under diagnosed globally^3,4^; early identification and reducing exposure to risk factors are important strategies to prevent dementia in the general population^5^.

Anticholinergic (AC) agents can block the acetylcholine (Ach) activity in both central and peripheral nervous systems^6^. The most commonly used ACs are tricyclic antidepressants, first-generation antihistamines, and bladder antimuscarinics^7^. Several studies in western countries have suggested that ACs might affect cognition, thereby increasing the risk of dementia among users^7–9^. Therefore, ACs are recommended to be avoided in frail and older adults. However, most of these studies showed limited correlation between the use of ACs for the central nervous system and dementia development. It is unclear whether the increased risk of dementia observed in these studies was caused by ACs specifically or with interaction of other medications used for co-existing brain disorders. Moreover, antimuscarinics may affect bladder function at the efferent or afferent axis. They serve as antagonists of the muscarinic AC receptor and operate on the post-junctional excitatory receptors in detrusor muscles^10^. There are considerable differences in brain penetration between the bladder antimuscarinics and ACs used for the central nervous system disorders. It is uncertain whether the urogenital use of antimuscarinic ACs would increase the risk of developing dementia due to the apparent pharmacodynamic differences between urogenital and central nervous ACs. For further investigation, we aimed to study the correlation between the exposure of bladder antimuscarinics and the risk of developing subsequent dementia. We used data from a nationwide, population-based database in Taiwan to analyze their possible relationships.

## Methods

### Data resource

The dataset used in this study was derived from the National Health Insurance Research Database (NHIRD) in Taiwan, which covers approximately 99% of the entire population of 23 million people in Taiwan. The Longitudinal Health Insurance Database (LHID) includes all original claims data and registration files from 2000 to 2013 for one million individuals randomly sampled from the Registry for Beneficiaries of the NHIRD program in 2000 in Taiwan. This database has been validated by several studies^11–13^, to prove the correct coding of different diseases. This study was approved by the Institutional Review Board of China Medical University and the Hospital Research Ethics Committee (IRB permit number: CMUH-104-REC2-115) and is in compliance with institutional guidelines. Written informed consent from patients was waived due to low risk, and the study was approved by the institutional IRB of China Medical University and the Hospital Research Ethics Committee.

### Study subjects

In this case–control study, we aimed to examine the effects of bladder antimuscarinics on the development of dementia. Study subjects comprised patients with dementia coded with ICD-9-CM 290, 294.1, and 331.0, and diagnosed by a neurologist or a general physician in the medical care system of Taiwan during 2000–2013. The first diagnosed date of dementia was defined as the index date. Disease diagnosis in the LHID was defined according to the International Classification of Disease, Ninth Revision, Clinical Modification (ICD-9-CM). The controls were subjects without dementia during 2000–2013 and were randomly selected from the LHID.

We excluded individuals younger than 55 years and individuals with missing data of age and sex. Considering that such a short-term exposure to antimuscarinics was less likely to cause dementia, subjects who used antimuscarinics for less than 1 year were excluded. For each dementia case, we used a propensity score matching method and randomly selected two controls from the non-dementia group. The controls were assigned the same index year as their matched cases with respect to age, sex, comorbidities, and medications mentioned below.

### Exposure assessment and covariates

The use of bladder antimuscarinics was evaluated before the index date. For those who used ACs for at least 1 year before the index date, we obtained data of seven types of bladder antimuscarinics (oxybutynin, G04BD04; propiverine, G04BD06; tolterodine, G04BD07; solifenacin, G04BD08; trospium, G04BD09; darifenacin, G04BD10; fesoterodine, G04BD11) based on World Health Organization ATC codes^14^. Patients without any prescription of ACs during the study period were classified as AC non-users. The duration of AC use was categorized as medium (1–3 years), long (4–7 years), and prolonged (> 7 years) durations. The cumulative dose of AC use during the study period was quantified for each patient using the World Health Organization Defined Daily Dose (DDD)^14^, and graded as follows: non-use, low-dose (≤ 207), medium-dose (207–3271), and high-dose (> 3271) users.

Several modifiable risk factors are shared among patients with dementia^1,5^, and we additionally adjusted for the effects of occurrence of various cardiovascular diseases to predispose dementia among the subjects. Therefore, we adjusted pre-existing comorbidities including hypertension (ICD-9-CM code 401–405, A260, and A269), stroke (ICD-9-CM code 430–437, and A29), transient ischemic attack (ICD-9-CM code 435.9), subarachnoid hemorrhage (ICD-9-CM code 852.0), coronary heart disease (ICD-9-CM code 414.00, 414.05, 414.8, and 414.9), heart failure (ICD-9-CM code 428.0), atrial fibrillation (ICD-9-CM code 427.9), hyperlipidemia (ICD-9-CM code 272.0–272.4), and diabetes mellitus (ICD-9-CM code 250 and A181). Moreover, anxiety (ICD-9-CM code 300.0), depression (ICD-9-CM code 296.2, 296.3, 296.82, 300.4, 309.0, 309.1, and 311), bipolar disorder (ICD-9-CM code 296.0, 296.1, 296.4, 296.5, 296.6, 296.7, 296.8, and 296.89), schizophrenia (ICD-9-CM code 295 and A211), severe learning difficulties (ICD-9-CM code 319), cognitive decline (ICD-9-CM code 311), asthma (ICD-9-CM code 493), chronic obstructive pulmonary disease (ICD-9-CM code 490–496), and renal disease (ICD-9-CM code 403.01, 403.11, 403.91, 404.02, 404.03, 404.12, 404.13, 404.92, 404.93, V42.0, V45.1, V56.x, and 790) were included and adjusted^15,16^.

Furthermore, we adjusted for the potentially confounding effects of other drugs, including aspirin, nonsteroidal anti-inflammatory drugs, antihypertensives, statins, anxiolytics, hypnotics, antidepressants, and anti-Parkinson’s disease and antipsychotic medications. Treatment with these drugs before the index date was evaluated as a part of the analysis.

### Statistical analysis

Propensity score matching was used to optimize comparability between the dementia and non-dementia groups using a non-parsimonious multivariable logistic regression model, with dementia as the dependent variable. Age, sex, comorbidities, medications, and index year were used as independent variables to match cases between the two groups. Descriptive statistics for the cases of dementia and non-dementia groups were reported, including demographic characteristics, comorbid disease, and medications. The standardized difference was used to test the differences in continuous and categorical matching variables. A standardized mean difference of ≤ 0.10 indicates a negligible difference between the groups.

We used conditional logistic regression to assess the risk of dementia associated with bladder antimuscarinics. The odds ratio (OR) and 95% confidence interval (CI) for dementia were calculated and subsequently adjusted for covariates including age, sex, comorbidities, and medications. The covariates adjusted for in the analytical models were listed as adjusted OR (aOR). To assess the dose–effect relationship, we analyzed the risks of dementia according to the cumulative DDD of bladder antimuscarinics (≤ 207 DDD, 207–3271 DDD, and > 3271 DDD) relative to non-users and stratified by 1–3, 4–7, and > 7 exposure years. We used SAS statistical software (Version 9.4 for Windows; SAS Institute, Inc., Cary, NC, USA) for data analysis. Results with a P-value of less than 0.05 were considered to be statistically significant.

## Results

**Figure 1:**
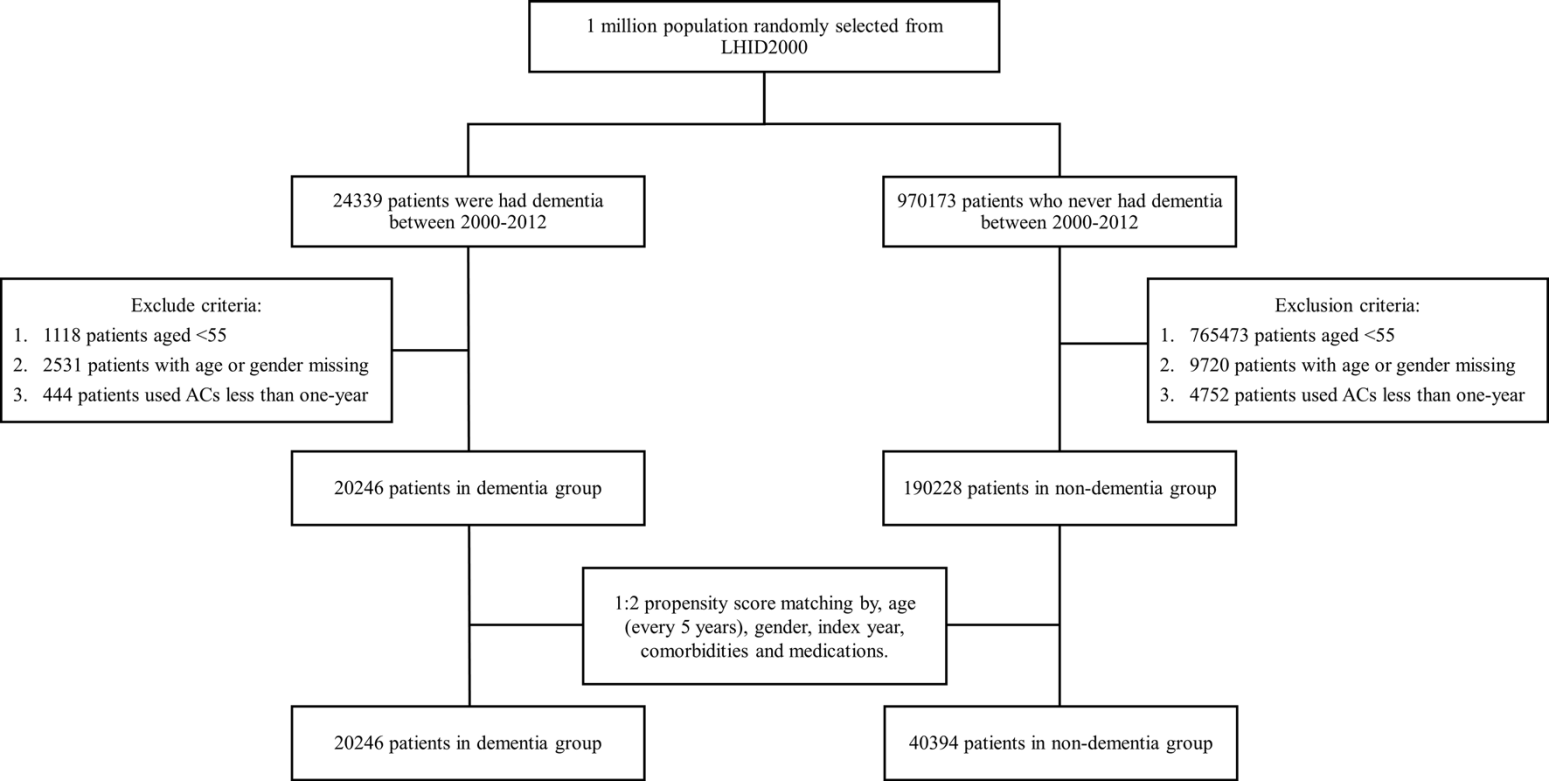
Flow chart for establishing antimuscarinic use and comparison cohorts using the National Health Insurance Research Database (NHIRD).

Table 1 shows the demographic and clinical characteristics of the study population. A total of 20,246 patients with dementia and 40,394 patients without dementia were enrolled in this study between January 1, 2000 and December 31, 2013 in the propensity score-matched population. The mean (SD) age was 77.3 (8.54) and 77.3 (10.3) years in the dementia and non-dementia groups, respectively. Among the subjects, females aged 75 to 84 years were dominant (Table 1). After propensity score matching, distribution of age, sex, comorbidities, and medications did not significantly differ between the groups. The detailed flow chart for the identification of the study subjects is shown in Fig. 1.

**Table 1:** Characteristics of patients with and without dementia and comparison between baseline and during follow-up.

Characteristic	Original population^a^, no. (%)		Standardized difference^§^	PS matched population^b^, no. (%)		Standardized difference^§^
	Dementia cohort	Non dementia cohort		Dementia cohort	Non dementia cohort	
	(n = 20,690)	(n = 194,980)		(n = 20,246)	(n = 40,394)	
**Sex**						
Female	10,849 (52.4)	93,169 (47.8)	0.093	10,625 (52.5)	20,626 (51.1)	0.028
Male	9841 (47.6)	101,808 (52.2)	0.093	9621 (47.5)	19,768 (48.9)	0.028
**Age at diagnosis of dementia**						
55–64	1898 (9.17)	86,719 (44.5)	0.869	1869 (9.23)	5388 (13.3)	0.13
65–74	5672 (27.4)	55,493 (28.4)	0.023	5566 (27.5)	11,030 (27.3)	0.004
75–84	9280 (44.8)	35,567 (18.2)	0.598	9055 (44.7)	14,533 (36.0)	0.179
85–94	3609 (17.4)	14,042 (7.20)	0.315	3533 (17.4)	7721 (19.1)	0.043
≥ 95	231 (1.12)	3159 (1.62)	0.043	223 (1.10)	1722 (4.26)	0.197
Age at diagnosis of dementia (mean, SD)^†^	77.3 (8.53)	68.8 (10.5)	0.888	77.3 (8.54)	77.3 (10.3)	0.001
**Comorbidity**						
Hypertension	10,007 (48.4)	58,313 (29.9)	0.385	9781 (48.3)	20,635 (51.1)	0.055
Stroke	4858 (23.5)	26,496 (13.6)	0.257	4750 (23.5)	9983 (24.7)	0.029
Transient ischemic attack	417 (2.02)	1830 (0.94)	0.089	407 (2.01)	808 (2.00)	0.001
Subarachnoid hemorrhage	130 (0.63)	510 (0.26)	0.055	129 (0.64)	251 (0.62)	0.002
Coronary heart disease	1675 (8.10)	7950 (4.08)	0.169	1639 (8.10)	3319 (8.22)	0.004
Heart failure	1765 (8.53)	7794 (4.00)	0.188	1721 (8.50)	3395 (8.40)	0.003
Atrial fibrillation	35 (0.17)	148 (0.08)	0.027	35 (0.17)	67 (0.17)	0.002
Hyperlipidemia	6476 (31.3)	35,008 (17.9)	0.314	6326 (31.2)	13,099 (32.4)	0.025
Diabetes	6992 (33.8)	39,287 (20.1)	0.311	6827 (33.7)	14,271 (35.3)	0.034
Anxiety	4093 (19.7)	21,192 (10.8)	0.249	4000 (19.7)	8285 (20.5)	0.019
Depression	3418 (16.5)	16,673 (8.55)	0.242	3337 (16.5)	6913 (17.1)	0.017
Bipolar disorder	488 (2.36)	2240 (1.15)	0.092	475 (2.35)	970 (2.40)	0.004
Schizophrenia	735 (3.55)	3564 (1.83)	0.107	717 (3.54)	1476 (3.65)	0.006
Severe learning difficulties	142 (0.69)	711 (0.36)	0.045	140 (0.69)	290 (0.72)	0.003
Cognitive decline	906 (4.38)	3939 (2.02)	0.134	882 (4.36)	1755 (4.34)	0.001
Asthma	5525 (26.7)	29,949 (15.3)	0.281	5394 (26.6)	11,518 (28.5)	0.042
COPD	10,404 (50.3)	61,864 (31.7)	0.384	10,177 (50.3)	21,551 (53.3)	0.062
Renal disease	2211 (10.7)	10,814 (5.55)	0.189	2161 (10.7)	4498 (11.1)	0.015
**Medication**						
Aspirin	16,415 (79.3)	109,091 (55.9)	0.516	16,049 (79.2)	32,063 (79.4)	0.003
Nonsteroidal anti-inflammatory drugs	20,322 (98.2)	177,372 (90.9)	0.325	19,884 (98.2)	39,606 (98.1)	0.012
Antihypertensives	19,244 (93.0)	141,644 (72.6)	0.561	18,823 (92.9)	37,731 (93.4)	0.017
Statin	5839 (28.2)	33,062 (16.9)	0.272	5704 (28.2)	11,265 (27.9)	0.006
Anxiolytic	19,226 (92.9)	151,847 (77.8)	0.436	18,798 (92.8)	37,251 (92.2)	0.024
Hypnotic	10,175 (49.1)	55,308 (28.3)	0.437	9960 (49.2)	19,701 (48.8)	0.008
Anti-depressants drug	9721 (46.9)	25,319 (13.0)	0.657	8231 (40.7)	11,460 (28.4)	0.261
Anti-Parkinson’s disease drug	4177 (20.2)	22,563 (11.6)	0.583	3767 (18.6)	3400 (8.42)	0.301
Anti-psychotic drug	12,885 (62.3)	76,063 (39.0)	0.702	12,616 (62.3)	21,428 (53.0)	0.188

*PS* propensity score, *COPD* chronic obstructive pulmonary disease.

^a^All comorbidities and medications before ps matching.

^b^All comorbidities and medication were after ps matching.

*P-value using chi-square for the comparisons between with and without fetal adverse.

^†^Average age using Wilcoxon rank-sum test for verification.

^§^A standardized mean difference of ≤ 0.10 indicates a negligible difference between the cohorts.

Table 2 presents the association between bladder antimuscarinics and the risk of developing dementia. After adjusting for potential confounders, antimuscarinic users exhibited a 2.46-fold increased risk of dementia compared with that in non-users (95% CI = 2.22–2.73). With respect to comorbidities, subjects with hypertension (aOR = 0.93, 95% CI = 0.89–0.98), asthma (aOR = 0.91, 95% CI = 0.89–0.97), and COPD (aOR = 0.86, 95% CI = 0.82–0.90) exhibited a lower risk of developing dementia. As for medication use, patients using aspirin, nonsteroidal anti-inflammatory drugs, statin, anxiolytics, and hypnotics exhibited a lower risk of developing dementia. On the contrary, those using anti-depressants drug, anti-Parkinson's disease drug, and anti-psychotic drug showed a higher risk of developing dementia (Table 2).

**Table 2:** Risk of dementia with prior use of bladder antimuscarinic drugs, other medications, and comorbidities.

Variable	N	Dementia	Crude OR (95%CI)	P-value	Adjusted OR (95%CI)*	P-value
**Bladder antimuscarinic drugs**						
Non-use	57,833	19,212	**1 (reference)**		**1 (reference)**	
Use	2807	1034	1.17 (1.08–1.26)	< 0.0001	2.46 (2.22–2.73)	< 0.0001
**Comorbidity**						
*Hypertension*						
No	30,224	10,465	**1 (reference)**		**1 (reference)**	
Yes	30,416	9781	0.89 (0.86–0.92)	< 0.0001	0.93 (0.89–0.98)	0.0064
*Stroke*						
No	45,907	15,496	**1 (reference)**		**1 (reference)**	
Yes	14,733	4750	0.93 (0.89–0.97)	0.0007	1.00 (0.94–1.06)	0.99
*Transient ischemic attack*						
No	59,425	19,839	**1 (reference)**		**1 (reference)**	
Yes	1215	407	1.00 (0.89–1.13)	0.93	1.05 (0.88–1.25)	0.57
*Subarachnoid hemorrhage*						
No	60,260	20,117	**1 (reference)**		**1 (reference)**	
Yes	380	129	1.02 (0.82–1.26)	0.81	1.03 (0.76–1.38)	0.82
*Coronary heart disease*						
No	55,682	18,607	**1 (reference)**		**1 (reference)**	
Yes	4958	1639	0.98 (0.92–1.04)	0.6	1.00 (0.91–1.09)	0.98
*Heart failure*						
No	55,524	18,525	**1 (reference)**		**1 (reference)**	
Yes	5116	1721	1.01 (0.95–1.07)	0.68	1.07 (0.98–1.17)	0.11
*Atrial fibrillation*						
No	60,538	20,211	**1 (reference)**		**1 (reference)**	
Yes	102	35	1.04 (0.69–1.57)	0.84	1.11 (0.70–1.76)	0.62
*Hyperlipidemia*						
No	41,215	13,920	**1 (reference)**		**1 (reference)**	
Yes	19,425	6326	0.94 (0.91–0.98)	0.003	1.02 (0.97–1.08)	0.27
*Diabetes*						
No	39,542	13,419	**1 (reference)**		**1 (reference)**	
Yes	21,098	6827	0.93 (0.89–0.96)	< 0.0001	0.99 (0.95–1.04)	0.95
*Anxiety*						
No	48,355	16,246	**1 (reference)**		**1 (reference)**	
Yes	12,285	4000	0.95 (0.91–0.99)	0.03	1.03 (0.97–1.10)	0.31
*Depression*						
No	50,390	16,909	**1 (reference)**		**1 (reference)**	
Yes	10,250	3337	0.95 (0.91–1.00)	0.05	1.00 (0.94–1.07)	0.83
*Bipolar disorder*						
No	59,195	19,771	**1 (reference)**		**1 (reference)**	
Yes	1445	475	0.97 (0.87–1.09)	0.67	0.95 (0.81–1.13)	0.62
*Schizophrenia*						
No	58,447	19,529	**1 (reference)**		**1 (reference)**	
Yes	2193	717	0.96 (0.88–1.06)	0.48	0.96 (0.83–1.11)	0.62
*Severe learning difficulties*						
No	60,210	20,106	**1 (reference)**		**1 (reference)**	
Yes	430	140	0.96 (0.78–1.17)	0.71	0.94 (0.70–1.25)	0.67
*Cognitive decline*						
No	58,003	19,364	**1 (reference)**		**1 (reference)**	
Yes	2637	882	1.00 (0.92–1.08)	0.94	1.03 (0.92–1.15)	0.53
*Asthma*						
No	43,728	14,852	**1 (reference)**		**1 (reference)**	
Yes	16,912	5394	0.91 (0.87–0.94)	< 0.0001	0.91 (0.86–0.97)	0.004
*COPD*						
No	28,912	10,069	**1 (reference)**		**1 (reference)**	
Yes	31,728	10,177	0.88 (0.85–0.91)	< 0.0001	0.86 (0.82–0.90)	< 0.0001
*Renal disease*						
No	53,981	18,085	**1 (reference)**		**1 (reference)**	
Yes	6659	2161	0.95 (0.90–1.00)	0.08	0.95 (0.88–1.02)	0.18
**Medication**						
*Aspirin*						
Non-use	12,528	4197	**1 (reference)**		**1 (reference)**	
Use	48,112	16,049	0.99 (0.95–1.03)	0.76	0.88 (0.84–0.92)	< 0.0001
*Nonsteroidal anti-inflammatory drugs*						
Non-use	1150	362	**1 (reference)**		**1 (reference)**	
Use	59,490	19,884	1.09 (0.96–1.23)	0.16	0.82 (0.72–0.95)	0.008
*Antihypertensives*						
Non-use	4086	1423	**1 (reference)**		**1 (reference)**	
Use	56,554	18,823	0.93 (0.87–0.99)	0.04	0.81 (0.75–0.87)	< 0.0001
*Statin*						
Non-use	43,671	14,542	**1 (reference)**		**1 (reference)**	
Use	16,969	5704	1.01 (0.97–1.05)	0.45	0.96 (0.92–1.00)	0.04
*Anxiolytic*						
Non-use	4591	1448	**1 (reference)**		**1 (reference)**	
Use	56,049	18,798	1.09 (1.02–1.16)	0.005	0.84 (0.78–0.90)	< 0.0001
*Hypnotic*						
Non-use	30,979	10,286	**1 (reference)**		**1 (reference)**	
Use	29,661	9960	1.01 (0.98–1.05)	0.32	0.83 (0.80–0.86)	< 0.0001
*Anti-depressants drug*						
Non-use	40,949	12,015	**1 (reference)**		**1 (reference)**	
Use	19,691	8231	1.73 (1.66–1.79)	< 0.0001	1.60 (1.54–1.67)	< 0.0001
*Anti-Parkinson's disease drug*						
Non-use	53,473	16,479	**1 (reference)**		**1 (reference)**	
Use	7167	3767	2.48 (2.36–2.61)	< 0.0001	2.22 (2.11–2.34)	< 0.0001
*Anti-psychotic drug*						
Non-use	26,596	7630	**1 (reference)**		**1 (reference)**	
Use	34,044	12,616	1.46 (1.41–1.51)	< 0.0001	1.29 (1.24–1.34)	< 0.0001

*OR* odds ratio.

*Adjusted for age, sex, all comorbidities, all medications, other anticholinergic drugs.

Table 3 presents the association between the cumulative DDD of bladder antimuscarinics and the risk of dementia by stratification according to the exposure to antimuscarinics. In patients who had been taking antimuscarinics for less than 4 years before the index date, an increased DDD was proportionally associated with the increased risk of developing dementia (aOR = 2.23, 95% CI = 1.12–4.44 for ≤ 207 DDD; aOR = 2.35, 95% CI = 0.87–6.32 for 207–3271 DDD; aOR = 12.8, 95% CI = 5.15–32.1 for > 3271 DDD) compared with that in the controls. In individuals exposed for 4–7 years, those who used ≤ 207 DDD (aOR = 2.82, 95% CI = 1.68–4.75), 207–3271 DDD (aOR = 2.23, 95% CI = 1.10–4.53), and > 3271 DDD (aOR = 1.90, 95% CI = 0.94–3.81) of antimuscarinics did not exhibit the trend of proportional increase in the risk of developing dementia. In patients with an exposure duration greater than 7 years, only those taking antimuscarinics at 207–3271 DDD (aOR = 1.19, 95% CI = 1.00–1.41) presented an equal risk of developing dementia compared with that in the controls (Table 3).

**Table 3:** Risk of dementia associated with cumulative use of bladder antimuscarinic drugs among study patients.

Exposure category	Study patients, no (%)			OR (95% CI)			
	Case patients	Controls		Unadjusted	P-value	Adjusted	P-value
**Exposure in the 1 to 3 years before index date**							
Patients, no.	1085	9493		NA		NA	
**Cumulative use (TSDDs)**							
Non-use	1062 (97.8)	9334 (98.3)		**1 (reference)**		**1 (reference)**	
≤ 207	10 (0.92)	96 (1.01)		0.91 (0.47–1.76)	0.79	2.23 (1.12–4.44)	0.02
207–3271	5 (0.46)	45 (0.47)		0.97 (0.38–2.46)	0.96	2.35 (0.87–6.32)	0.09
> 3271	8 (0.74)	18 (0.19)		3.90 (1.69–9.00)	0.001	12.8 (5.15–32.1)	< 0.0001
**Exposure in the 4 to 7 years before the index date**							
Patients, no.	1766	3980		NA		NA	
**Cumulative use (TSDDs)**							
Non-use	1706 (96.6)	3883 (97.6)		**1 (reference)**		**1 (reference)**	
≤ 207	31 (1.76)	43 (1.08)		1.64 (1.03–2.61)	0.03	2.82 (1.68–4.75)	< 0.001
207–3271	15 (0.85)	25 (0.63)		1.36 (0.71–2.59)	0.34	2.23 (1.10–4.53)	0.02
> 3271	14 (0.79)	29 (0.73)		1.09 (0.57–2.08)	0.77	1.90 (0.94–3.81)	0.07
**Exposure for more than 7 years before the index date**							
Patients, no.	17,395	26,921		NA		NA	
**Cumulative use (TSDDs)**							
Non-use	16,490 (94.8)	25,496 (94.7)		**1 (reference)**		**1 (reference)**	
≤ 207	256 (1.47)	444 (1.65)		0.89 (0.76–1.04)	0.14	1.07 (0.89–1.29)	0.42
207–3271	325 (1.87)	466 (1.73)		1.07 (0.93–1.24)	0.3	1.19 (1.00–1.41)	0.04
> 3271	324 (1.86)	515 (1.91)		0.97 (0.84–1.11)	0.69	1.03 (0.87–1.23)	0.66

*OR* odds ratio.

*Adjusted for age, sex, all comorbidities, all medications, and other anticholinergic drugs.

Table 4 presents the duration (years) of dementia identified in the dementia group and presents the year of study entry in the non-dementia group. We calculated the duration of exposure in both groups. The mean (SD) duration of exposure was 5.87 (3.96) and 5.93 (3.47) years in the dementia and non-dementia groups (Table 4).

**Table 4:** Number of patients identified and duration of exposure.

	Group				P-value
	Dementia cohort		Non-dementia cohort		
	N	%	N	%	
**Year of entry study**					0.04
2000	1189	5.87	2274	5.63	
2001	1105	5.46	2060	5.1	
2002	1103	5.45	2112	5.23	
2003	1129	5.58	2267	5.6	
2004	1374	6.79	2766	6.85	
2005	1368	6.76	2787	6.9	
2006	1454	7.18	2876	7.12	
2007	1524	7.53	2989	7.4	
2008	1479	7.31	2904	7.19	
2009	1638	8.09	3243	8.03	
2010	1676	8.28	3300	8.17	
2011	1743	8.61	3457	8.56	
2012	1790	8.84	3966	9.82	
2013	1674	8.27	3393	8.4	
**Duration of exposure, years**					
Mean (SD)	5.87 (3.96)		5.93 (3.47)		0.23

## Discussion

In this retrospective nation-wide population-based case–control study, we noted that the risk of dementia increased 2.46-fold in Taiwanese patients aged 55 years or older who had been previously using bladder antimuscarinics for 1 year or more. Specifically, the risk proportionally increased with increasing dosage in patients taking antimuscarinics for less than 4 years. Richardson et al. reported that dementia development was associated with an increased use of antidepressant, urological, and anti-Parkinson agents^6^. They suggested that prior exposure to ACs of up to 20 years before the diagnosis of incident dementia could be detected. However, in our study, we noted that dementia development might be associated with an increasing dose of antimuscarinics for medium exposure duration. We believe that antidepressants, antihistamines, and bladder antimuscarinics should be started at different ages in patients. A medium exposure duration (< 4 years) would be related to less death claims in elderly subjects on antimuscarinics than in those with long or prolonged use in this study. We carefully adjusted the possible confounding effects from central nervous system disorders and ACs used for purpose other than urogenital organs in the study design. To our knowledge, this is the first study to report the dose–response effects on dementia development in patients using bladder antimuscarinics for less than 4 years in an Asian population.

We further observed a different relationship between the risk of dementia development and the duration of antimuscarinics exposure than that in other studies. The risk of dementia development seemed to decrease by time in patients on a high dose of antimuscarinics (> 3271 DDD). Those on antimuscarinics and aged 75–84 years seemed to be resistant to a higher risk of developing dementia. It implied a higher mortality rate for the first few years in aged patients with urogenital disorders. Once they crossed the high-risk period of urogenital disorders, the very aged survivors show a trend to resist developing another disorder. As in any observational study, the positive association that we observed between bladder antimuscarinics and dementia risk should be considered a part of numerous factors involved in dementia patho-mechanism. The directionality of the association can only be hypothesized by current data. To maintain the quality of life of patients, we suggest that primary caregivers should not limit the use of antimuscarinics for urogenital disorders before the symptoms of dementia are observed in the patients.

In this study, we analyzed the correlations between seven bladder antimuscarinics (oxybutynin, propiverine, tolterodine, solifenacin, trospium, darifenacin, and fesoterodine) and the subsequent dementia development. There are five subtypes (M_1_–M_5_) of muscarinic ACh receptors, and the M2 and M3 muscarinic Ach receptors are the major receptors that mediate smooth muscle contraction, proliferation, and remodeling of the bladder^17,18^. In contrast, evidence from recent postmortem human brain studies have implicated the involvement of the M_1_ muscarinic Ach receptors in various psychiatric disorders^19,20^. Some studies have further demonstrated the dominant functional distribution of the M_1_ muscarinic receptors for ACh uptake in the human brain^21,22^. Our study results implied that bladder antimuscarinics might possibly produce subtle effects on M_1_ activity, in addition to their known antagonistic effects on the M_2_ and M_3_ receptors. Because of the potential effect of bladder antimuscarinics on the M1 muscarinic receptor, bladder antimuscarinics has been supposed to cause dementia development. Furthermore, we cannot completely rule out the role of other subtypes of muscarinic receptors besides the M_1_ receptor that exhibits less expressed in the brain, and the brain penetration of various bladder antimuscarinics might be different. More laboratory studies could help clarify the detailed mechanism of different antimuscarinics and expression of different ACh subtypes in dementia development.

Globally, approximately 47 million people suffer from dementia with an estimated global cost of 818 billion US dollar in 2015, and the patient number would triple by 2050^1^. Older adults who develop dementia are less likely to return to their ordinary lives than those who do not. The mechanisms of dementia development are too complicated to be fully understood in older adults with co-existing chronic disorders. First, older adults with bladder disorders might have sedentary lifestyles with poor sleep and personal hygiene or even with alcohol abuse and drug addiction. Sedentary lifestyles would potentially increase the risk of dementia development^23,24^. Second, bladder disorders often exist with local or systemic inflammation to associate with an increased risk of dementia development^15,16^. It is difficult to design a study that can distinguish the effects of inflammatory disorders from that of antimuscarinics on dementia development. However, the relationship between the risk of dementia and the dose of bladder antimuscarinics provides supports our hypothesis^25^. These results indicate that bladder antimuscarinics increase predisposition to dementia development.

The major limitation of this study was that dementia cases diagnosed using the ICD-9-CM coding system are often underestimated^26^. Although the NHI program covers nearly 99% of Taiwanese citizens and guarantees equality of access to medical services for everyone throughout the country, some dementia cases might be outside the scope of our study. With a higher rate of dementia diagnosis in older adults with bladder disorders, there might be some patients who did not receive bladder antimuscarinics before the incident dementia diagnosis. Second, we could not directly contact the patients because their identities were anonymized in the accessible LHID. Therefore, we could not analyze all confounding factors for dementia development within the patients’ families or the psychological burden on patients. Third, the poor adherence to bladder antimuscarinics in patients was another potential limitation of this study. However, our study demonstrates a statistically significant increase in the risk of dementia development in patients using bladder antimuscarinics. These results highlight the need to further explore bladder antimuscarinics as a predisposing factor for dementia development.

## Conclusions

Taiwanese patients aged 55 years or more undergoing treatment with bladder antimuscarinics exhibited a higher risk for dementia development. Additional studies in other countries are required to determine whether this result is valid worldwide.

## Data Availability

The original data are available at NHI, and we are not allowed to release despite reasonable application.
